# ‘Getting Better’—Is It a Feasible Strategy of Broad Pan-Antiherpesviral Drug Targeting by Using the Nuclear Egress-Directed Mechanism?

**DOI:** 10.3390/ijms25052823

**Published:** 2024-02-29

**Authors:** Julia Tillmanns, Jintawee Kicuntod, Josephine Lösing, Manfred Marschall

**Affiliations:** Institute for Clinical and Molecular Virology, Friedrich-Alexander University of Erlangen-Nürnberg (FAU), 91054 Erlangen, Germany; jul.tillmanns@fau.de (J.T.);

**Keywords:** human pathogenic herpesviruses, cytomegalovirus (HCMV), essential steps of viral replication, viral nucleocytoplasmic capsid egress, nuclear egress complex (NEC), core and multicomponent NEC extensions, novel antiviral drug targeting, NEC-directed mode of action, strategies of antiviral drug development

## Abstract

The herpesviral nuclear egress represents an essential step of viral replication efficiency in host cells, as it defines the nucleocytoplasmic release of viral capsids. Due to the size limitation of the nuclear pores, viral nuclear capsids are unable to traverse the nuclear envelope without a destabilization of this natural host-specific barrier. To this end, herpesviruses evolved the regulatory nuclear egress complex (NEC), composed of a heterodimer unit of two conserved viral NEC proteins (core NEC) and a large-size extension of this complex including various viral and cellular NEC-associated proteins (multicomponent NEC). Notably, the NEC harbors the pronounced ability to oligomerize (core NEC hexamers and lattices), to multimerize into higher-order complexes, and, ultimately, to closely interact with the migrating nuclear capsids. Moreover, most, if not all, of these NEC proteins comprise regulatory modifications by phosphorylation, so that the responsible kinases, and additional enzymatic activities, are part of the multicomponent NEC. This sophisticated basis of NEC-specific structural and functional interactions offers a variety of different modes of antiviral interference by pharmacological or nonconventional inhibitors. Since the multifaceted combination of NEC activities represents a highly conserved key regulatory stage of herpesviral replication, it may provide a unique opportunity towards a broad, pan-antiherpesviral mechanism of drug targeting. This review presents an update on chances, challenges, and current achievements in the development of NEC-directed antiherpesviral strategies.

## 1. Introduction

The family of *Herpesviridae* is characterized by a linear double-stranded DNA genome and a comparatively large size of membrane-enveloped particles comprising 100–300 nm [1]. All herpesviruses share a lifelong persistence within the host with extended latency periods and strongly reduced gene expression, followed by intermittent phases of reactivation causing recurrent symptoms [2,3]. With infection rates spanning approx. 60–95% in cases of human cytomegalovirus (HCMV), herpes simplex virus type 1 (HSV-1), and varicella zoster virus (VZV), herpesvirus infections affect the majority of adults worldwide [4,5]. Herpesviruses can be divided into subfamilies based on their morphology, genetics, and biological properties [6,7,8,9,10,11]. *Alphaherpesvirinae* (*α*) include herpes simplex viruses type 1 and type 2 (HSV-1/-2) and varicella zoster virus (VZV); *Betaherpesvirinae* (*β*) includes the human cytomegalovirus (HCMV) as well as human herpesviruses types 6A, 6B, and 7 (HHV-6A, HHV-6B, HHV-7); and *Gammaherpesvirinae* (*γ*) includes Kaposi’s sarcoma herpesvirus (KSHV/HHV-8) and Epstein–Barr virus (EBV/HHV-4).

Within human pathogenic α-herpesviruses, VZV represents a major pathogen, which causes chickenpox (varicella) upon primary infection, followed by VZV persistence in a state of nonproductive latency in the nervous system of the immunocompetent host. Consequences of VZV reactivations are lesions known as shingles (zoster), which can cause severe neurological diseases, such as acute sequelae or persistent burning pain [12]. Representing the only approved vaccine against a human herpesvirus, recommended in Germany by the RKI since 2004, or internationally by the WHO, Zostavax^®^ (live attenuated) and Shingrix^®^ (subunit vaccine) prevent primary or secondary infection with VZV [13]. As far as clinically dominant β-herpesvirus infections are concerned, HCMV is mostly asymptomatic or associated with mild symptoms in immunocompetent individuals [14,15,16]. In immunocompromised patients, however, such as transplant recipients, cancer patients, and human immunodeficiency virus type 1 (HIV-1)-infected individuals [17,18], HCMV can provoke severe consequences. Importantly, congenital HCMV infection (cCMV) is considered as the most urgent medical problem to be addressed by the development of novel preventive remedies. Representing the far most frequent vertically transmitted viral pathogenic infection during pregnancy [19,20], HCMV causes a wide range of symptoms, in more than 25% of all infected babies if the cases of late-onset disease are included. Symptoms span from mild to severe or even life-threatening (with approx. 10% of stillbirths contained in the group of acutely symptomatic), and the main clinical problems manifest as hearing/vision loss, mental retardation, and microcephaly in the unborn [21,22,23]. Concerning major human γ-herpesvirus infections, EBV is extremely widespread in the adult human world population (>6 billion infected), and, especially in immunity-based risk constellations, is associated with various malignant tumors, including post-transplant B- and T-cell lymphoma, Hodgkin’s lymphoma, Burkitt’s lymphoma, and nasopharyngeal and gastric carcinoma [24,25,26].

While vaccination is only available against VZV, a number of antiviral drugs are in use against α- and β-herpesvirus infections. Most approved herpesviral therapeutics are nucleoside/nucleotide analogs or other compounds likewise affecting the viral genome replication. For HCMV, the gold standard is still ganciclovir (GCV) and its orally administrated, bioavailable prodrug valganciclovir (VGCV), representing two related acyclic guanosine analogs. Both are activated through monophosphorylation by one of the respective nucleoside-converting herpesviral kinases (i.e., thymidine kinase of α-herpesviruses, or protein kinase of β- and γ-herpesviruses), thus exerting a certain specificity for herpesvirus-infected cells [27,28]. Further inhibitors of herpesviral genome synthesis are the nucleotide analog cidofovir (CDV) and the pyrophosphate analog foscarnet (FOS), used as a second line of therapy for GCV-resistant infections [29,30]. Frequently occurring side effects of viral genome replication inhibitors include nephrotoxicity, myelotoxicity, and anemia [31,32,33]. Two recently added HCMV drugs are letermovir (LMV), which inhibits the viral terminase, and maribavir (MBV), which affects the viral kinase activity, both clinically approved during the last years. Of note, however, subunit pUL56 of the terminase complex, which is important for the processing and encapsidation of newly synthesized viral genomes [34], may acquire LMV-directed resistance mutations. LMV is currently limited to prophylactic use in hematopoietic stem cell transplant recipients [35,36]. Also, MBV treatment can lead to resistance formation based on gene mutations of the viral kinase pUL97 [37,38,39,40], so the clinically urgent need to resolve drug resistance issues still persists. Although MBV proved to be associated with a lower incidence of severe and treatment-limiting adverse events than standard GCV therapy [41], another limitation of MBV is the lack of an option to combine MBV treatment with GCV. Due to the fact that the target of MBV, pUL97, is necessary for the activating phosphorylation of GCV, an antagonistic drug interaction is predictive, which has actually also been confirmed experimentally [42]. Nevertheless, MBV is highly special in another aspect, as it represents the first approved kinase inhibitor in the entire field of antiviral therapy. Beyond that, by targeting the NEC-associated kinase pUL97, MBV also represents the first identified drug acting through a herpesviral nuclear egress-directed mechanism. Whether MBV, LMV, or any other novel drug in development may become helpful in cCMV therapy or prevention is still in question [43,44].

**Figure 1:**
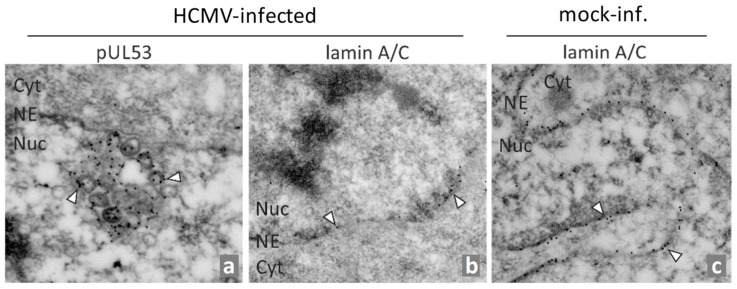
Immunogold EM analysis of HCMV-infected primary human fibroblasts (HFFs). HFFs were infected with HCMV AD169 for 6 days (**a**,**b**) or remained uninfected (**c**), before cells were fixed, subjected to sectioning and immunogold staining for EM analysis. Immunostaining was performed for human nuclear lamin A/C or viral pUL53 as indicated. Individual gold particles were exemplarily marked by arrowheads. NE, nuclear envelope; Cyt, cytoplasm; Nuc, nucleus (modified from [54]).

Thus, there is a need to identify new targets and treatment options in order to complement the currently available drugs and to improve anti-HCMV and further antiherpesviral treatment options. The process of nuclear capsid egress provides a rate-limiting step during viral replication, so it has been considered as a promising target, and has actually been experimentally validated in various aspects [45]. Since the diameter of newly assembled capsids (approximately 130 nm) prevents their exit via nuclear pores, a multifaceted, fine-regulated process is necessary for their nucleocytoplasmic transport [46,47,48,49]. A key element of the HCMV-specific nuclear egress complex (NEC) is the core NEC, consisting of the two viral proteins pUL50 and pUL53. This core NEC unites at least three major functions of nuclear egress, namely the recruitment of various NEC-associated effector proteins (multicomponent NEC), the reorganization of the nuclear lamina and membranes (nuclear rim distortion), and the interaction with nuclear capsids for nucleocytoplasmic transition (NEC–capsid docking). The individual stages of this process have been investigated by a variety of methodological approaches [50] and, in particular, electron microscopy (EM)-based techniques revealed highly interesting details. For HCMV, approaches of confocal imaging and immunogold EM labeling deciphered preferred sites of nuclear capsid egress, termed as lamina-depleted areas [51,52,53], as well as a pronounced intranuclear association of pUL53 with viral capsids [54,55], either in proximity or at a distance from the nuclear rim (Figure 1, image a). This accumulation of viral capsids was associated with a typical thinning of the lamina (Figure 1b, compared to c), as effected by site-specific phosphorylation and subsequently induced lamin A/C reorganization [51,56,57].

**Figure 2:**
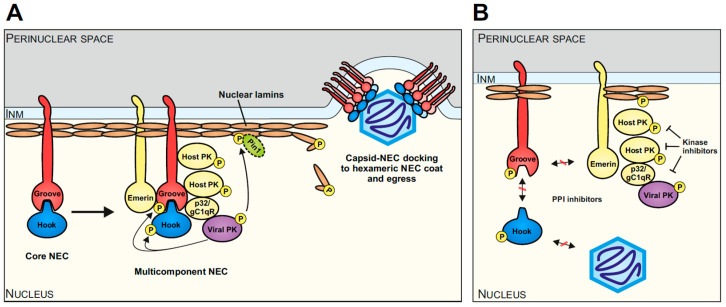
Schematic overview of herpesviral nuclear egress, highlighting the example of HCMV and current options of antiviral targeting. (**A**) The core NEC, consisting of a hook (pUL53 of HCMV) and groove (pUL50 of HCMV), recruits several other viral and cellular proteins, such as emerin, p32/gC1qR, host protein kinases (PKs), the viral PK (pUL97 of HCMV), Pin1, and possibly further effector proteins to phosphorylate and modify the nuclear lamina. The resulting reorganization of the nuclear lamina together with the hexameric arrangement of the core NEC heterodimers at the INM facilitates the egress of the viral capsid into the perinuclear space (modified from [48]). (**B**) Diverse targeting options of the NEC include the inhibition of initial core NEC heterodimerization, capsid docking, and interactions with further components of the multicomponent NEC (indicated via red-crossed interaction arrows). Kinase inhibitors such as MBV have already demonstrated strong antiviral activity by targeting the nuclear egress (indicated via blocked arrows).

The herpesviral core NEC generally plays a central role in the multistep regulation of nuclear egress and recruits the multicomponent NEC. Interestingly, this extended complex also includes a number of host proteins, which fulfill important roles in the regulation of viral nuclear egress. In the case of HCMV, the multicomponent NEC involves emerin, p32/gC1qR, protein kinase C (PKC), cyclin-dependent kinases 1 and 2 (CDK1, CDK2), possibly further host CDKs/kinases, the viral kinase vCDK/pUL97, and the peptidylprolyl cis/trans isomerase Pin1 (Figure 2A [50,52,55,58,59,60,61]). The site-specific phosphorylation of lamins leads to a Pin1-dependent disruption of the nuclear lamina that allows de novo assembled capsids to reach the inner nuclear membrane (INM) [51]. The process, encompassing assembly, nucleocytoplasmic transition, and capsid maturation, is conserved in structure and function between α-, β-, and γ-herpesviruses [40,48,50,62,63,64]. As the herpesviral NEC recruits a number of different protein–protein interactions (PPIs), regulatory activities, and transport-specific functions, the options for drug-mediated targeting are broad and diversified. So far, the most interest has been paid to the analysis and development of small molecules interfering with NEC-specific PPI and kinase activities (Figure 2B).

**Figure 3:**
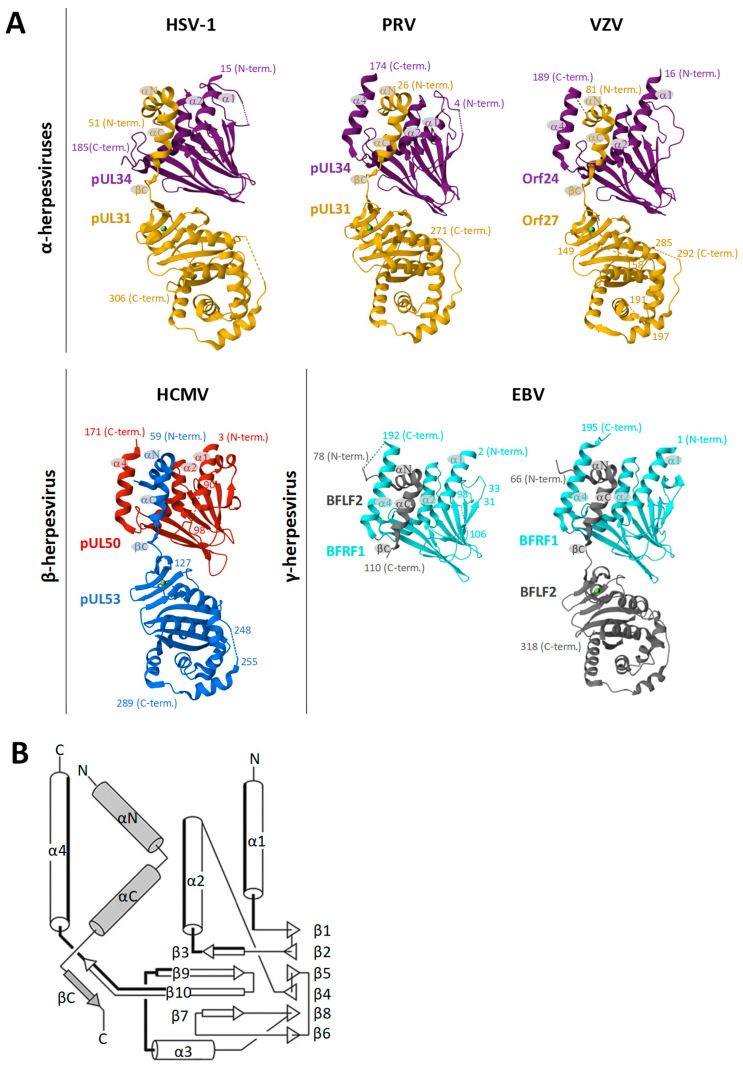
Summarized depiction of crystallization-based 3D structures of herpesviral core NECs resolved so far. (**A**) Groove proteins are depicted in violet, red, or cyan, with hook proteins in yellow, blue, or grey. Secondary structure elements involved in heterodimerization are indicated. Crystal structures were obtained from the Protein Data Bank (PDB)—HSV-1, 4ZXS [69]; PRV, 5E8C [72]; VZV, 7PAB [68]; HCMV, 5D5N [66]; EBV, 6T3Z [65]—and recently published including the globular domain of BFLF2 7T7I [73]. Additional valuable structural information on NECs has been provided by several more studies [69,70,72,74,75,76,77]. (**B**) Common topology plot visualizing the interacting secondary structure elements of the core NEC: hook segments indicated in grey, groove counterparts highlighted in bold [65].

As central elements of the NEC, HCMV pUL50 and pUL53, or their homologs of the other herpesviruses, play a crucial role as a binding platform. Both proteins adopt a globular fold with mixed secondary structure elements (Figure 3). While pUL50 is located within the INM via a transmembrane domain (TMD), pUL53 comprises a classical nuclear localization signal (NLS) [65,66]. pUL53 carries a hallmark element by its N-terminal hook-like extension, consisting of two consecutive α-helices followed by a short β-strand (Figure 3A). This hook structure contributes around 80% of the interaction surface with the groove-like structure of pUL50, mainly composed of four α-helices, i.e., α1, α2, and α4 adjacent to a loop segment formed by α3, as linked to the beta-sheet β9 [48,65,67] (Figure 3B). Based on core NEC secondary structural elements, the nucleoplasmic pUL53 interacts in a hook-into-groove-like principle with its integral membrane protein counterpart pUL50, in a manner identical between all three herpesviral subfamilies [65,66,68]. Compared to the nearly fully conserved crystal structures of the core NECs (Figure 3) [48,69,70], the amount of sequence conservation, as measured by amino acid identity, is relatively limited [71].

So far, a number of mechanistic modes have been described that may be utilized for developing NEC-directed drugs [45]. These include the blocking of core NEC formation, the interference with activities of the multicomponent NEC (such as NEC-associated kinase, isomerase, or transport activities), the interference with capsid docking to the NEC, or the blocking of membrane-specific activities during primary envelopment [45]. As the first NEC-associated prototype inhibitor directed to viral kinase pUL97, MBV exerts a strong nuclear egress-inhibitory activity. Its inhibitory mode of action (MoA) concerns the pUL97-mediated phosphorylation of nuclear lamin and core NEC proteins as well additional multicomponent factors. This situation supports the strategy of a multifactorial option of inhibition, as based on a herpesviral nuclear egress-specific mechanism [22].

## 2. The Multifaceted Aspects of NEC-Directed Antiviral Drug Targeting—An Updating Review

### 2.1. Investigational Tools That Utilize the Conditional Expression of Viral NEC Proteins

#### 2.1.1. Illustration of the Rate-Limiting Step of Herpesviral Replication by the Generation of Mutant NEC Versions

During recent decades, a number of novel MoAs have been investigated in defining antiviral target proteins and in various aspects of antiherpesviral research. These did not only lead to the understanding of essential functions throughout the entire herpesviral replication cycle, but also to the molecular characterization of the regulatory process of nuclear egress. In this proceeding, very valuable investigational tools were generated that led to a very clear validation of the NEC as a rate-limiting and drug-accessible step of herpesviral replication. Many approaches used conditionally expressed viral NEC proteins to illustrate their functional importance [45]. As a general conclusion, the importance of the core NEC was recognized, i.e., a highly conserved component, for the efficiency of replication of α-, β-, and γ-herpesviruses. Interestingly, however, there have also been reports that describe NEC-independent alternatives of viral egress and the identification of NEC-deficient mutants showing residual maintenance of viral replication. As a consequence, the essential and nonessential functional properties of herpesviral core NECs have been discussed. For two α-herpesviruses, herpes simplex virus type 1 (HSV-1) and pseudorabies virus (PrV), this issue has been specifically addressed. Data strongly suggested that pUL31 of HSV-1 and PrV (hook proteins) is not absolutely essential for nuclear egress [78,79,80]. Surprisingly, the deletion of ORF-UL31 from the HSV-1 or PrV genomes did not lead to a complete block of viral replication, but instead to a relative decrease in viral loads and a partial deficiency in the production of enveloped particles. This phenotype could be restored by the use of pUL31-complementing cells. Very similar findings were also obtained for the deletion of α-herpesviral gene sequences encoding ORF-UL34 (groove proteins; [81,82,83]). In these studies, a striking result was obtained by an extensive passaging of the PrV ORF-UL31 or ORF-UL34 deletion mutants, which led to functional variants, termed pass mutants, almost producing wild-type titers [84,85]. Notably, similar mutations were detected in various viral proteins which appeared to be associated with a virus-induced disintegration of the nuclear envelope (NE), a phenomenon described as nuclear envelope break-down (NEBD). In these cases, NEBD seemed to serve as an alternative to the regulated nuclear egress that facilitated, at least in part, the basal level of progeny production of these mutant viruses. Further experimental indications pointed to mitosis-related processes possibly involved in this herpesvirus-induced NEBD. Likewise for EBV, deletion mutants in the coding sequences for nuclear egress proteins BFRF1 and BFLF2 were described [86,87]. In these experimental models of NEC deletion, a low-level replication of the recombinant viruses was demonstrated, specifically showing strong defects in the efficiency of nuclear egress and primary envelopment. Thus, although these assessments of herpesviral NEC functionality showed that viral NEC proteins may not be absolutely essential, at least not for basal levels of viral replication, their regulatory role is strictly linked with a wild-type-like, high efficiency of nuclear egress. 

The studies of our group focused on HCMV and addressed the assignment of an essential functional role to the HCMV core NEC by characterizing the ΔUL50 virus, a recombinant HCMV lacking ORF-UL50. Surprisingly, this ΔUL50 virus, as reconstituted on pUL50-complementing cells (ΔUL50C) and used as a stock virus for further infection experiments, was found impaired but not completely blocked in replication as seen with non-complementing cells. Interestingly, we detected a strong decrease in the production of mature C-type capsids in the absence of pUL50 expression, with a nuclear accumulation of immature A-type capsids. Interestingly, two different versions of ΔUL50 particles could be compared, i.e., those produced by pUL50-complementing cells (ΔUL50C) and those by non-complementing cells (ΔUL50N); the latter, of course, were obtained at much lower quantities. A basic finding of this comparative study was that the ΔUL50N particles, derived from non-complementing cells, exhibited a substantially stronger replicative defect than ΔUL50C particles [54]. This defect became even more apparent when performing a gradient purification of the low quantities of ΔUL50N. Remarkably, the proteomic composition of noninfectious particles, derived from the distinct ΔUL50 preparations (i.e., ΔUL50C and ΔUL50N), did not differ to a relevant extent. However, experiments confirmed that the packaging of viral genomes into the capsids of ΔUL50N was massively disturbed in the absence of pUL50. Thus, it could be concluded that the ΔUL50N particles lacked the normal portion of C-type capsids and encapsidated, infectious virions. As a consequence, this set of experimentations demonstrated that HCMV pUL50 was not found to be irreplaceably essential for nuclear egress, but it definitely possesses a crucial regulatory role in viral egress and maturation that also includes the packaging of viral genomes. 

Based on the previously described core NEC deletion mutant ΔUL50, in the context of pUL50-complementing cells, the system was extended, allowing for investigations of both core NEC proteins in parallel. This sophisticated recombinant HCMV ΔUL50-ΣUL53 was designed for the individual and conditional expression of pUL50 and/or pUL53 in the context of HCMV replication [88]. Based on the BAC HCMV AD169 ΔUL50 [89], the ORF of pUL50 was deleted but pUL50 expression can be complemented by doxycycline (dox)-inducible pUL50 overexpressing cells. On the other hand, pUL53, expressed from the viral genome, is N-terminally fused to a destabilizing domain based on the cellular FKBP12 protein (ddFKBP), as linked by a short six-amino-acid long spacer region. The entire fusion protein is stabilized by the addition of a small ligand, Shield-1, whereas its absence leads to the proteasomal degradation of ddFKBP::pUL53. Through the individual addition of dox for pUL50 expression and/or Shield-1 for pUL53 stabilization, different HCMV replication settings with both, only one, or none of the core NEC proteins are comparable. The conditional expression as well as a physiological behavior of the core NEC proteins, including nuclear colocalization and interaction properties, were proven. However, as previously discussed, the core NEC heterodimer is of main importance for efficient particle maturation and replication in general, leading to a low residual replication efficiency of HCMV ΔUL50-ΣUL53, especially if both components are missing. This limitation can be overcome by normalizing investigated antiviral effects to their solvent control, allowing comparisons between the different infectious settings. Taken together, HCMV ΔUL50-ΣUL53 represents a very valuable tool for the functional characterization of this heterodimer in general as well as of core NEC affecting small molecules and peptides [88].

#### 2.1.2. The ‘Shared-Hook’ Strategy: Addressing Mutant-Specific Cross-Viral NEC Interaction Patterns to Prepare a Platform for Broadly Active NEC Inhibitors

**Figure 4:**
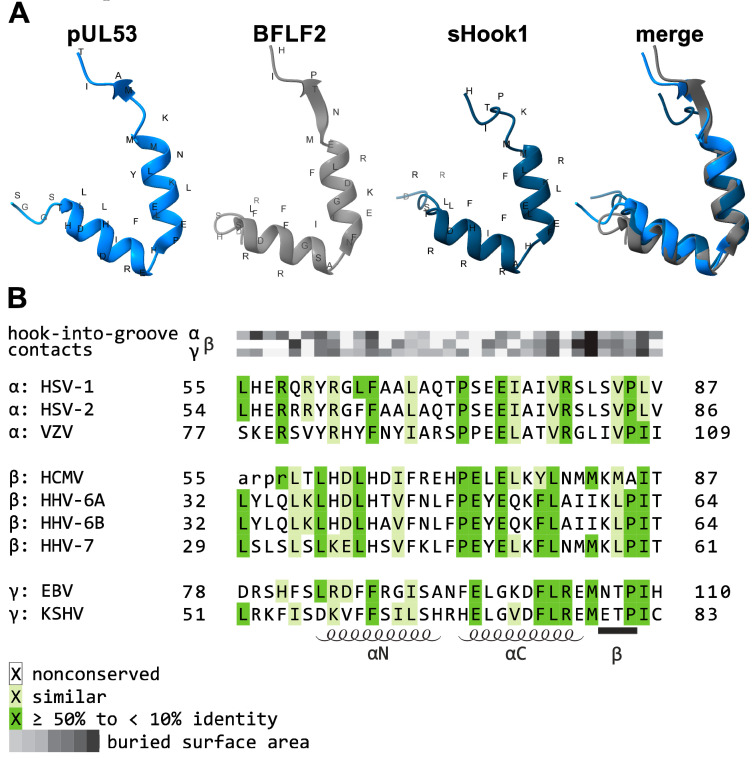
Principle of the shared-hook strategy and sequence alignment of homologous hook proteins. (**A**) This depiction illustrates our concept of the chimeric merge between important interaction-determining contact points, in order to generate variants of hook proteins with a potential shared-hook binding activity. Such gain-of-activity of specific mutants facilitates the binding to various groove proteins of different herpesviral subfamilies. The wild-type hook structures of HCMV pUL53 (amino acids 55–87) and EBV BFLF2 (amino acids 78–110), as well as the structure of a previously published shared-hook construct, termed sHook1 [67], are shown from left to right. sHook1 is a combination of significant contact amino acids or interaction properties between pUL53 and BFLF2 that are crucial for NEC formation. The ‘merge’ illustration confirms that the predicted structure of sHook1 fits between those of pUL53 and BFLF2, thus converging the structures (i.e., note in the merge panel that the dark-blue helical hook extension of sHook1 is interspaced between the two parental hooks of pUL53 and BFLF2 in light blue and grey, respectively). The crystal structures of pUL53 (PDB: 6T3X) and BFLF2 (PDB: 6T3Z) were previously determined experimentally [65]. The three-dimensional structure of sHook1 was predicted on the basis of software Colabfold v1.5.5 (AlphaFold2 using MMseqs2). (**B**) Amino acid alignment, highlighting conserved amino acids and amino acids representing contact interfaces. Increasing levels of sequence conservation are indicated by darker shades of green in the alignment. The buried surface area at hook-into-groove contact positions in α-, β-, and γ-herpesviruses is indicated by grey squares. Darker shades of grey indicate a larger buried surface. Lower-case letters mark those residues that were not resolved in the crystal structure. The elements of the secondary structure are depicted schematically below the alignment. HSV-1, herpes simplex virus type 1; HSV-2, herpes simplex virus type 2; VZV, varicella zoster virus; HCMV, human cytomegalovirus; HHV-6A, human herpesvirus 6A; HHV-6B, human herpesvirus 6B; HHV-7, human herpesvirus 7; EBV, Epstein–Barr virus; KSHV, Kaposi’s sarcoma-associated herpesvirus (Figure 4 represents a refined illustration that refers to [48]).

Previous studies have repeatedly reported that the interaction between core NEC proteins in a cross-viral manner is possible and is restricted to the respective herpesviral subfamilies [71,90,91,92]. On this basis, the conserved interface of the core NEC binding structures led to the postulation of a ‘shared-hook’ binding strategy (Figure 4A) [67]. This concept is based on sequence-structure predictions that are intended to generate hook constructs capable of binding to various groove proteins of other α-, β-, and/or γ-herpesviruses (‘shared-hook’ binding). These extended, cross-viral NEC interaction patterns may provide a valuable starting point for the development of broad-spectrum inhibitory small molecules.

For the design of these adapted hook constructs, the primary sequences of the underlying hook proteins were carefully examined (see concept in Figure 4). Important interaction characteristics were compared, and those sequence stretches predicted to contribute to the hook-into-groove interface were conceptually combined into potential shared-hook variants (Figure 4A). Particularly, the hook-into-groove interaction-determining contact amino acids were extracted from either two or each of the three subfamilies in order to define common consensus stretches, also according to their buried surface areas. In addition, amino acids with a high degree of identity across all herpesviral subfamilies were included (Figure 4B, dark green).

Thus, our research group successfully generated such shared-hook constructs and demonstrated cross-viral interaction using different PPI methods. However, interaction did not necessarily result in the complete complementation of all functions of the naive hook protein. Mutations may likewise result in a partial loss-of-function, primarily by reducing the interaction affinity. To investigate the actual functionality of these mutated hook proteins, which has a proven shared-hook binding activity, a complementation system using recombinant viruses could be utilized. Especially, the HCMV AD169-GFP ΔUL53 virus strain, lacking the coding sequence for the hook protein, was used to infect HFF cell populations that conditionally expressed shared-hook variants of pUL53 via a dox-inducible promotor.

The use of this system demonstrated that mutated hook proteins, although active in core NEC binding, may be limited in their ability to functionally complement the viral ΔUL53 defect. To examine the complementation ability of mutated hook constructs in detail, either qPCR of infectious supernatants or a quantitation of green fluorescent protein (GFP)-positive cell counts were used as readout systems for the determination of HCMV AD169-GFP ΔUL53 replication kinetics. Previous analyses have shown that sHook1-Flag, one of the first shared-hook variants obtained by our research group, interacted with both its HCMV counterpart and the EBV groove protein, thus demonstrating cross-viral interaction. However, a complementation analysis indicated that sHook1-Flag was not as effective as the wild-type hook protein, pUL53, in supporting HCMV replication. On this basis, further shared-hook variants are continuously being analyzed in the approach to identify functional complementation. Thus, the shared-hook system is considered valuable in the assessment of functionally active variants, and it is noteworthy to emphasize that the hook-into-groove interaction was recognized as a conserved principle across all herpesviral subfamilies. This aspect is pertinent in the understanding of cross-viral core NEC interaction, so that the knowledge about residues that determine the shared-hook target site may facilitate the future development of broadly acting inhibitory compounds. These should not only target a virus-specific NEC but may enable a broader targeting option, in ideal terms, against several or all human pathogenic herpesviruses.

### 2.2. Antiviral Block of Nuclear Egress Functions by the Intracellular Overexpression of NEC-Inhibitory Hook Fragments

**Figure 5:**
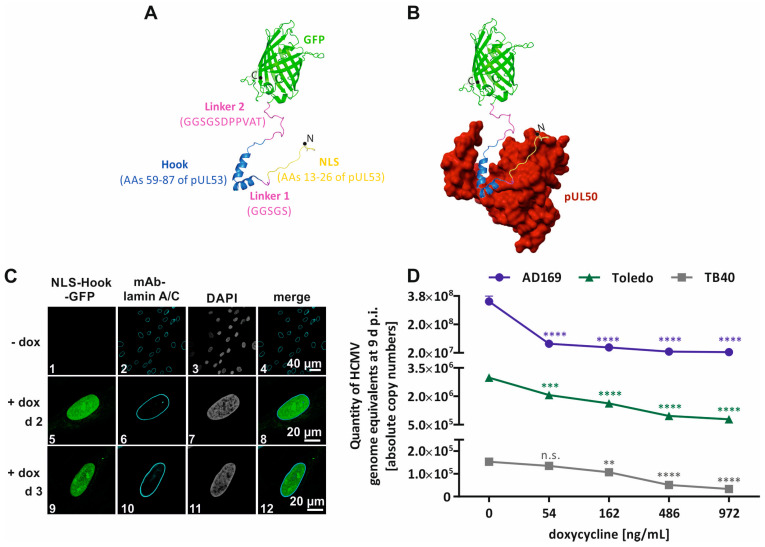
Inducible intracellular expression of NLS-Hook-GFP as an NEC-interfering fragment illustrating anti-HCMV activity. (**A**) Construct of NLS-Hook-GFP depicts the NLS sequence of pUL53 (yellow), the hook fragment of pUL53 (navy blue; PDB entry 6T3X [65,100]), the reporter GFP protein (green; PDB entry 1EMA [101]) and two linker segments (magenta). (**B**) A model of pUL50 (red; PDB entry 5D5N [66]) is predicted to interact with NLS-Hook-GFP. (**C**) Intracellular expression of NLS-Hook-GFP is able to be induced via addition of dox. (**D**) The replication levels of various HCMV strains were plotted against concentrations of dox [98]. ANOVA with Dunnett’s multiple comparison test was applied to evaluate statistical significance in comparison to non-induced cells: ****, *p* ≤ 0.0001; ***, *p* ≤ 0.001; **, *p* ≤ 0.01; n.s., *p* > 0.05.

The NEC has been validated as a promising target for antiherpesviral strategies via numerous approaches [88,92,93,94,95,96,97,98]. The latest proof-of-concept was established, termed ‘NLS-Hook-GFP’, in order to assess the efficacy of HCMV replication upon an intracellular expression of the pUL53 hook fragment [98]. A lentiviral gene transfer procedure was utilized to produce a primary fibroblast (HFF) population, in which the intracellular expression of a nuclear localization signal (NLS, amino acids 13–26 of pUL53) fused to the HCMV-specific pUL53 hook fragment (Hook, amino acids 59–87 of pUL53) and the GFP reporter (Figure 5A) could be induced by the addition of dox. Over the period of 2–3 days (d) post-induction, the recombinantly expressed NLS-Hook-GFP could be observed to occur at its typical localization in the nucleoplasm via confocal imaging (Figure 5C, images 5, 8, 9, and 12; see the lamin A/C nuclear envelope signal in images 6 and 10 and the DAPI counterstaining of nuclei in images 7 and 11). The specific NEC interaction of this expressed construct was identified by an in vitro assembly assay as previously described [71,97,98]. The data revealed that NLS-Hook-GFP specifically interacted with the HCMV groove protein (pUL50; Figure 5B); however, it did not exhibit an interaction with the HCMV parental hook protein (pUL53). Moreover, the inducible NLS-Hook-GFP was also able to interact with the closely related groove protein of murine CMV (pM50), but it did not bind to the remotely related groove homologs, Orf24 of VZV or BFRF1 of EBV. In addition, the authors further investigated the interaction of HCMV core NEC proteins (pUL50–pUL53) in the presence of the NLS-Hook-GFP construct, utilizing an in vitro assembly assay. The signals derived from the coimmunoprecipitation (CoIP) of transfection-derived pUL50 and pUL53, in the presence of an inducible hook fragment, illustrated a slight decrease in pUL50–pUL53 interaction in comparison to a combination of pUL50 and pUL53 alone. In addition to this, a similar in vitro assembly assay was performed with proteins from HCMV-infected HFFs. As an important result, the protein signals from the CoIP of both pUL50 and pUL53 could be detected in the presence of intracellular NLS-Hook-GFP expression. Notably, the protein signals of HCMV core NEC proteins in the lysate controls declined along the increasing expression levels of the hook fragment. At this point, the results derived from plasmid-transfected and HCMV-infected cells showed the specific binding of NLS-Hook-GFP to the cytomegaloviral core NEC proteins. Furthermore, the authors investigated whether the intracellular expression of NLS-Hook-GFP may intervene in the viral replication, and thereby demonstrate antiviral activity against HCMV replication, by qPCR measurement and Western blot (Wb) analysis. The quantity of HCMV genome equivalents was diminished in the increasing expression levels of the inducible Hook construct over a period of 9 d post-infection (i.e., three rounds of viral replication). The positive signals of pUL53 and pp28 from the Wb analysis were prominently reduced as compared to non-induced cells [98]. The replication levels of HCMV strain AD169 under NLS-Hook-GFP expression were decreased compared to a similar tendency when using a second laboratory strain, TB40, and a clinically relevant strain, Toledo (Figure 5D). Moreover, the confocal imaging of immunofluorescence staining demonstrated an alteration of the typical localization of pUL53 on the nuclear rim, resulting in a dot-like structure under induced expression of NLS-Hook-GFP in HCMV-infected cells. In addition, the data from an established nuclear egress assay (NEA) illustrated decreased levels of viral genome equivalents in the cytoplasmic fraction in correlation to the higher intracellular expression of NLS-Hook-GFP [89,98,99]. The formation of a cytoplasmic virion assembly complex (cVAC), which was investigated by pp150 immunofluorescence staining, was likewise dispersed under high expression levels of inducible hook fragments in HCMV-infected HFFs [97,98]. Taken together, this proof-of-concept strategy suggests that the inducible NLS-Hook-GFP expression had a specific inhibitory effect on the HCMV core NEC, which limited viral nuclear egress. These findings support the idea that the core NEC could be a novel target for discovering new antiherpesviral strategies and candidate drugs.

### 2.3. Identification of First NEC-Directed Small Molecules Comprising a Pronounced Level of Antiviral Activity

During the course of validating herpesviral NECs as suitable and effective targets of a novel type of antiviral drug discovery, several reports described methodological approaches, relevant mechanistic insight, and NEC targeting concepts to prepare antiviral research and compound screenings [71,88,92,93,95,96,97,102,103,104,105]. The approach to target a viral core NEC structure is challenging in the way that, first of all, it demands the identification of specific PPI inhibitors instead of conventional enzymatic inhibitors. In the study by Schnee et al. [92], a protein complementation assay (PCA), using β-lactamase fragment fusions of interacting proteins, was applied to define the specific properties of the herpesviral core NEC components. The murine wild-type MCMV pM50 and pM53, and mutants thereof, were compared in complexation with homologous pairs of HCMV, HSV-1, pseudorabies virus (PRV), EBV, and murine herpesvirus 68 (MHV-68). Cross-complementation was found to be positive only within the same herpesvirus subfamily. Moreover, the HCMV homologs rescued replication-defective MCMV genomes lacking one of the core NEC genes. Using this PCA, the authors also discussed an initial approach to screen for NEC-specific PPI inhibitors or NEC mutants conferring such inhibitor resistance [92].

Concerning the broader search for NEC inhibitors in the fields of α-, β-, and γ-herpesviruses, not many reports of successful outcomes have been published. This stands in contrast to the massively increased molecular understanding of NEC targeting requirements, as achieved through the description of high-resolution 3D structures and additional information on NEC biochemistry [65,66,68,69,72,75]. Current approaches have recently been reported based on the utilization of covalently target-binding small molecules (see Section 2.4 [88,95]).

To this end, a very relevant approach was described based on the use of the Prestwick Chemical Library^®^, which appeared to adopt a way-paving character. These works were based on an in vitro binding inhibition assay of the pUL50–pUL53 interaction, performed to screen the Prestwick Chemical Library^®^ that comprised 1520 chemically and pharmacologically diverse compounds mainly approved by the FDA for the treatment of various diseases [93,97]. The approach identified two hits, i.e., merbromin (MBM) and verteporfin, exhibiting inhibitory activity against the HCMV pUL50–pUL53 interaction in the nanomolar range [97]. In a second step, the inhibitory effect of MBM was confirmed in several HCMV infection experiments, and the first findings showed that this compound inhibits the formation of the HCMV core NEC at the nuclear rim. Additional characteristics that illustrated the NEC-based inhibitory activity of this drug have then been elaborated. Due to its mercury content, however, MBM did not appear as an ideal drug candidate by itself, but might serve as a blueprint for further developments. In conclusion, several experimental points of evidence argued for the strong potential of core NEC-directed antivirals. Moreover, the existing knowledge about molecular details of the core NEC, including crystal structures of α-, β-, and γ-herpesviral core NECs, and the continuous improvement of computer-based methods may substantially enhance this developmental approach.

**Figure 6:**
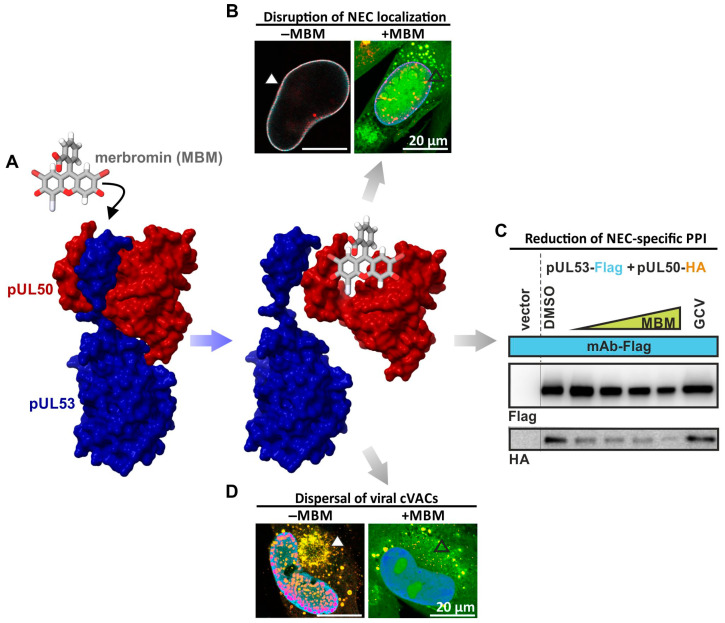
Summarized depiction of mechanistic properties of the prototypic NEC-inhibitory small molecule merbromin (MBM). (**A**) Chemical structure of MBM. (**B**) A merge of all signals was captured using a confocal microscope after performing immunofluorescence staining for viral pUL53 (red; Alexa Fluor 555) and cellular lamin A/C (far-red; Alexa Fluor 647) and counterstaining of the auto-fluorescent drug (MBM; green). MBM disrupts the localization of pUL53 on the nuclear rim (filled arrow head) in HCMV-infected primary fibroblasts, which can be detected in dot-like structures (empty arrow head). (**C**) The expression plasmids of pUL53-Flag and pUL50-HA were used for the single transient transfection of 293T cells and harvested at 2 days post-transfection for the preparation of total lysates. Combinations of these lysates were used for in vitro assembly in the presence of MBM, as indicated above the immunoblots. DMSO (solvent control) and ganciclovir (GCV; antiviral reference drug) were used as potential inhibitors. After rotation at 4 °C overnight, the assembled samples were subjected to immunoprecipitation (IP) with mAb-Flag and subsequently analyzed in a standard SDS-PAGE/Wb procedure. The protein interaction of HCMV core NEC, pUL50–pUL53, decreases in the presence of higher concentrations of MBM. (**D**) MRC-5 cells were infected with recombinant TB40 UL32-GFP, prior to the treatment with DMSO or auto-fluorescent MBM (green). Cells were fixed and subjected to indirect immunofluorescence staining for the cVAC marker pUL32-GFP (yellow–red; Alexa Fluor 555) and IE1 (far-red; Alexa Fluor 647). The yellow–red signal of cVAC formation (filled arrow head) is dispersed after treatment with MBM (empty arrow head) [45,93,97].

In terms of addressing the mechanistic properties of MBM as a postulated HCMV NEC-specific PPI inhibitor, an in vitro NEC assembly assay was established [97]. Using this analysis system for measuring the HCMV core NEC interaction, it was shown that increasing concentrations of MBM (0, 1.25, 2.5, 5, and 10 μM) effected a pronounced MBM-mediated reduction in NEC in vitro assembly (Figure 6A), in which the anti-HCMV reference drug GCV proved to be ineffective. Moreover, MBM exerted a negative impact on nuclear rim formation of the core NEC and on viral nuclear egress activity in HCMV-infected cells (Figure 6B [93,97]). Also, MBM reduced the NEC-specific PPI in vitro (Figure 6C), and proved to be both compound- and target-specific, as no inhibition was seen for other compounds or other viral proteins. Especially, MBM effected a strong inhibition of HCMV infection in the low micromolar range, as demonstrated by various assay conditions and readouts (Figure 6D). This line of evidence confirmed the concept that an NEC-directed PPI inhibitor has a great potential as an antiviral candidate compound.

### 2.4. Covalently Target-Binding Warheads Can Possess an NEC-Directed Antiviral Potency with Amenability for Further Drug Optimization

**Figure 7:**
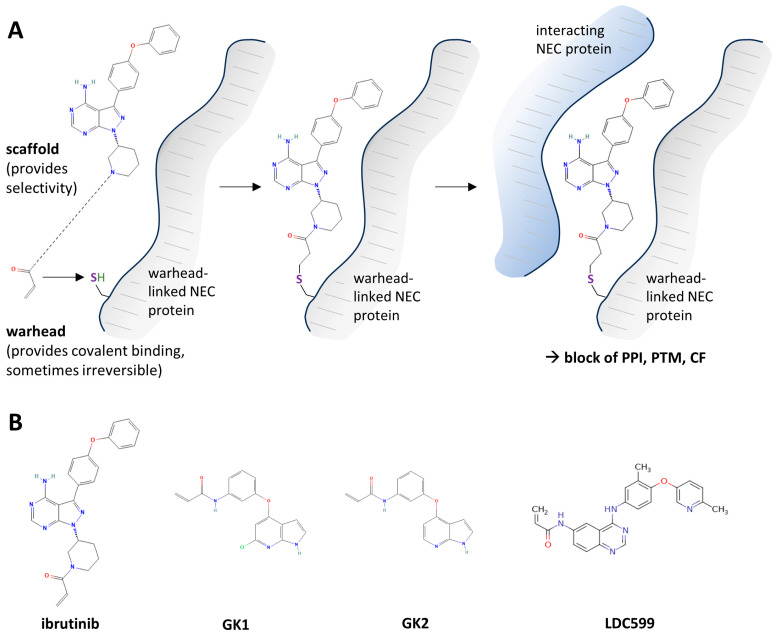
Covalent target binders, termed ‘warhead’ compounds, which possess a proven NEC-directed mode of anti-HCMV activity: principle of MoA and examples of so-far-analyzed compounds. (**A**) These covalently binding compounds act in a two-step process, with the drug scaffold providing a certain degree of target selectivity and bringing the warhead moiety into close proximity to its nucleophilic target residue. Subsequently, the α-β-unsaturated carbonyl group covalently binds its target residue, typically a cysteine, and covers the interaction surface of the two core NEC counterparts, thereby primarily affecting protein–protein interaction (PPI), but possibly also post-translational modifications (PTM) or conformational flexibility (CF). (**B**) Chemical structure of recently identified HCMV core NEC-inhibiting warhead compounds.

Due to large the PPI interface of the herpesviral core NEC, small molecules may not cover the entire binding surface and may be displaced during PPI activity by the interaction partner. To circumvent this problem, targeted covalent inhibitors represent a new antiviral concept [106]. Originally developed as kinase inhibitors, covalently binding inhibitors carry a specific functional group, so called warheads, e.g., an α-β-unsaturated carbonyl moiety. This electrophilic functionality can interact with an accessible nucleophilic residue at its biological target, normally a cysteine residue (Michael addition reaction) [107,108,109,110]. While the covalent target linkage of warheads is thereby achieved through the Michael addition reaction, binding specificity is mediated by the drug scaffold. Ultimately, the association with its target structure may acquire a scaffold-mediated specificity, which then provides close proximity for the covalent interaction (Figure 7A) [111,112]. Thus, the postulated chances of warheads to interfere with large-interface PPIs are given by their high-affinity covalent binding capacity, an aspired site-specific docking, and eventually by preventing drug displacement by the protein interaction partner. In addition to the effects exerted on the PPI level, warheads may likewise interfere with the targets’ post-translational modification (PTM) or conformational flexibility (CF; Figure 7A). In the case of the HCMV core NEC, computational analysis defined several cysteine residues, both in pUL50 and its counterpart pUL53, which were found partly located within the hook-into-groove interaction surface. Due to their exposed position within this interface, e.g., at amino acid Cys54 of pUL50, these identified cysteine residues represent promising candidates for the targeting approaches with covalent inhibitors [88]. Two recently published, independent studies have discovered covalently binding warhead compounds as a new class of antiviral agents that target the core NEC of HCMV (Figure 7B).

The source of our own investigations was a series of newly generated warhead-containing compounds (LDC599 and others), which were used for a first evaluation of putative antiviral and core NEC-inhibitory properties, as demonstrated by several different PPI- and infection-based methodological approaches [88]. For further investigations of the inhibitory potential of covalent binders, a selection of commercially available, clinically relevant warheads guided us to ibrutinib, an already approved drug for the treatment of B cell malignancies. In addition ibrutinib’s primary activity as a host-directed kinase inhibitor, the drug is proven to act as an experimental inhibitor of HCMV core NEC interaction. This inhibitory effect was most probably mediated through a secondary covalent binding activity to the viral NEC protein(s). In this context, it has to be emphasized that ibrutinib and related drugs were recognized as binders of a specific primary target (e.g., Bruton’s tyrosine kinase in case of ibrutinib), with maximized levels of high target IC_50_ and low SI values. Considering such determination of selectivity panels, however, secondary targets may never be excluded. Our focus was directed to the identification of these additional drug binding affinities, putatively directed to virus-specific target structures. By this route, the antiviral activity of warheads including ibrutinib was identified, primarily using HCMV-infected cells, at non-cytotoxic concentrations. Moreover, the study demonstrated a serial concentration-dependent inhibitory activity of ibrutinib in a split luciferase-based PPI evaluation assay (NanoBiT), as well as a drug-mediated disruption of the typical core NEC rim formation (confocal imaging). Ibrutinib was active against the HCMV-specific NEC, but not herpesviral NECs of other subfamilies. To further address this NEC-directed targeting effect of ibrutinib, a conditionally regulated core NEC expression system, based on recombinant HCMV ΔUL50-ΣUL53, was used for confirmation. In the absence of HCMV core NEC protein expression, ibrutinib comprised strongly reduced antiviral activity [88]. 

Shortly after this first report, a second study was published, confirming the covalent warhead inhibitor approach to target the HCMV pUL50–pUL53 heterodimer [95]. From a huge screening approach, using several different libraries, the authors identified two core NEC-affecting, acrylamide moiety-containing covalent binders, named GK1 and GK2, which only differed in a chlorine at the 7-azaindole of GK1, being a hydrogen on GK2. Both compounds exerted antiviral activity against HCMV, but lower activity against HSV-1, which was interpreted as an initial sign of virus specificity. The investigated compounds led to a moderate delocalization of pUL53 from the nuclear rim, which was less prominent compared to a positive control, i.e., the use of a known mutant of pUL53 (L79A [113]) that strongly reduced rim interaction with pUL50. In parallel, late viral gene expression, evaluated by pp28, showed partial inhibition upon GK1 treatment. As an important result, a modified version of GK2, lacking its acrylamide moiety, did not longer exhibit core NEC-blocking activity, confirming that this chemical group was important for PPI inhibition. Interestingly, the authors were able to define one important residue, i.e., Cys214 in pUL53, as the putative inhibitor docking site using mass spectrometry-based proteomic analysis. This cysteine was confirmed in its relevance by the generation of an HCMV C214S replacement mutant, which comprised resistance against GK1 treatment. On the other hand, however, this substitution did not significantly affect GK2 inhibition, thus indicating a complex mode of GK2 target binding. Interestingly, GK2 binding to Cys54 in pUL50 was additionally detected, which, however, only had a minor effect on core NEC interaction. Furthermore, several GK1 analogs with anti-core NEC activity, against so far unknown residues, might help to detect further target sites within pUL50 or pUL53 [95]. It should also be mentioned that the study by Chen et al. [95] supported the previously discovered NEC-directed effects of ibrutinib and merbromin [88,93,97], although it could not directly provide a confirmation, as using different methodological approaches. Nevertheless, these studies demonstrated the NEC-specific activity of warhead moiety-containing compounds, thus emphasizing the suitability of the approach. Notably, the drug scaffold, which carries the α-β-unsaturated carbonyl group, is intended to provide binding selectivity towards the target protein. At the current stage of investigations, i.e., by the use of the first discovered warheads possessing NEC-directed activity [88,95], these aspects of covalent binding efficacy, scaffold-mediated selectivity, and antiviral potency may be subject to the next steps of conceptual optimization. The approach may continuously benefit from the promising advantages already seen with covalent target binders; i.e., these may comprise a high binding affinity, low-dosage efficacy, extended durations of action, and a particularly efficient suitability in PPI inhibition [111,114]. In this regard, ibrutinib and GK1 may serve as a starting point for the further development of core NEC-specific, covalently binding prototype antivirals.

### 2.5. Design and Application of Synthetic NEC-Mimetic Cell-Penetrating Peptides (CPPs)

The peptide-based approach of interference with herpesviral core NEC formation represented another challenging topic of these novel options of antiviral targeting. In this case, the question had to be experimentally addressed as to whether the design of an NEC-binding synthetic peptide would ensure cellular uptake and the crossing of cell membranes. To this end, the previously reported amino acid sequences of cell-penetrating peptides (CPPs [94,115,116,117,118,119,120,121]) could be utilized to generate synthetic chimeras that included three different functions [94]: (i) a 29-mer peptide, representing the minimal binding fragment of HCMV pUL53 (α-helical N-terminal hook-like region) to its NEC counterpart pUL50 (groove), was synthetically fused with (ii) a small cationic or amphipathic CPP sequence, in connection with (iii) the autologous pUL53 nuclear localization signal (NLS, amino acids 18–27 [122]). These model peptides, termed NLS-CPP-Hook, were then analyzed for the specific properties of the inhibition of core NEC assembly and putative antiviral potential. The in vitro analysis of binding characteristics demonstrated a high-affinity interaction with pUL50. Moreover, the hook-derived peptides showed a pronounced NEC-interfering activity, at nanomolar concentrations, in terms of a competitive binding and blocking of the assembly of recombinant pUL50–pUL53 or the corresponding NEC peptides [65,94]. Even more relevant was the application of these peptides to cultured cells, especially to primary human fibroblasts infected with HCMV. Actually, it was considered as a very valuable step forward to see that NLS-CPP-Hook, and related peptides with fluorescent labels, was uptaken by cells and were able to associated with the viral NEC as determined by confocal imaging. Although the efficiency of yielding peptide-positive cells remained limited, it was possible to detect both cytoplasmic and nuclear signals of peptide uptake. Importantly, the inhibition of HCMV replication could be demonstrated by several different methodological approaches. The fusion peptide NLS-CPP-Hook showed activity in the delocalization of pUL53 from the nuclear rim, in the reduction of cells positive for viral replication, and in reduced quantities of virus progeny release [94]. The findings of this report illustrated, on the one hand, the antiviral efficacy of an application of synthetic NEC-mimetic peptides, and, on the other hand, the suitability of a herpesviral core NEC as a potential antiviral target. In the field of α-herpesviruses, Draganova et al. [96] identified synthetic peptides which were derived from the HSV-1 capsid protein pUL25. Experimental evidence was provided, using an in vitro model system based on giant unilamellar vesicles (GUVs), that these peptides were capable of inhibiting the membrane-budding activity of the viral NEC, considered as a correlated activity of viral nuclear egress. The study showed that the inhibitory ability of capsid-derived peptides depended on their propensity to form an α-helical structure. The results were interpreted as a basis for the development of an alternative class of inhibitors directed against nuclear egress.

### 2.6. Kinase Inhibitors Directed to Important NEC-Associated CDK and vCDK Activities

The association of herpesviral core NECs with additional controlling factors was one of the door-opening perceptions in the understanding of nuclear egress regulation. Of note, this aspect has already been addressed by the initial report of associated kinase activity required for NEC functionality. In the case of murine cytomegalovirus (MCMV), the recruitment of cellular kinases, specifically protein kinase C (PKC), was recognized as a crucial step to dissolve the nuclear lamina [123]. PKC was likewise found to be associated with the HCMV-specific NEC [59,60,124,125], the functional relevance of which has remained in question [57,125]. Although the experimental proof of a direct binding of host kinases to the herpesviral core NECs has not been successful for some time, the functional studies on NEC regulation strongly substantiated this statement (see numerous reviews: [45,48,50,62,64,126,127,128,129,130,131,132]). It became increasingly clear that, although the association of PKC could not be directly linked with functional importance, other host and viral kinases played a very crucial role in the regulatory phosphorylation of NEC proteins [125,133,134,135] and nuclear lamins [50,56,58].

In particular, the regulatory association of cyclin-dependent kinases (CDKs) types 1, 2, and 5 [99,125], as well as the viral ortholog HCMV vCDK/pUL97, was repeatedly demonstrated [61]. In particular, the role of HCMV pUL97 and related herpesviral kinases, such as EBV BGLF4 as well as HSV-1/-2 pUL13 and pUS3, moved into focus [56,58,61,136,137,138]. The distinct functional importance of HCMV pUL97 for the regulated efficiency of viral nuclear egress [22,139,140,141], and its direct association with viral capsids during the egress process [55], strongly fostered the validation of this kinase as a target for antiviral therapy. The pUL97-targeting drug MBV was thus clinically approved in 2021 by the U.S. FDA as the first kinase-specific herpesviral nuclear egress inhibitor [142]. Possibly, the further development of inhibitors of pUL97, derived from alternative chemical drug classes, may additionally broaden this option of antiviral interference [143,144,145,146,147]. In this context, it should also be emphasized that the few examples in which herpesviral multicomponent NECs have been deciphered in detail clearly point to even more options of the targeting of NEC-associated effector function. Notably, however, despite the common importance of NECs, the composition of the entire multicomponent NEC can profoundly differ between individual herpesviruses (for HCMV, see [51,61,148]; for MCMV, see [149]; for HSV-1, see [150]; for EBV, see [50,151]). In essence, the studies on NEC-associated factors, such as protein kinases, CDK and vCDK activities, and the great potential of kinase inhibitors, strikingly illustrate their importance for overall NEC functionality.

### 2.7. Further Mechanistic Modes to Interfere with Herpesviral Nuclear Egress

The herpesviral NEC has attracted the deep interest of researchers, since it represents a regulatory key position of viral replication and a putative target for novel antiviral strategies. Due to the fact that this NEC can be dissected into two functional units, namely the core structural platform consisting of a heterodimeric hexamer- and even oligomer-forming portion and a regulatory multicomponent extension made up of associated factors, this target reveals many faces. Currently, the mainly investigated options of NEC-related inhibition include PPI-interfering small molecules, peptides, antibodies, NEC fragments, and similar agents. In essence, all of these approaches are focused on the core NEC. When considering, however, the additional functional properties of NEC in terms of NEC-associated factor recruitment, nuclear lamina reorganization, and capsid interaction, even more targeting mechanisms come into play. Through treatments with selective inhibitors of NEC-associated protein kinases, such as viral kinase pUL97-directed MBV, the phosphorylation of nuclear lamins can be blocked. This site-specific phosphorylation, mainly concerning lamin A/C serine 22 [51,56], stands central in the process of the egress-typical reorganization of the nuclear envelope and the formation of lamina-depleted areas [51,52,57,105,123,152,153,154,155]. Of note, however, the introduction of a negative charge by phospho-modification is most probably not sufficient to induce such drastic structural changes. In case of HCMV, the phospho-serine 22-dependent binding of prolyl cis/trans isomerase Pin1 has been demonstrated, so an isomerization-triggered conformational change in nuclear lamin A/C is currently seen as the most probable driving force [50,51,52,156]. Concludingly, the application of Pin1 inhibitors may be additionally considered in terms of an antiviral NEC-associated targeting mechanism. Such a preventive approach may possibly be based on a combined drug application including two or more NEC-specific MoAs. It also appears plausible to take into account the options of developing blockers of capsid–NEC docking. This concept of a nuclear entrapment of viral capsids has also been substantially supported by mechanistic studies, e.g., those using viral mutants carrying defects in the core NEC proteins responsible for capsid binding [54,126,157,158,159]. Although this approach has not been clearly defined for antiherpesviral strategies, a capsid-specific inhibitor, lenacapavir, is presently under clinical investigation for therapeutic anti-HIV-1 activity [160]. And even more specifically, the option of utilizing encapsidation-interfering agents might be addressed. In this sense of interfering small molecules, their binding to herpesviral capsids, genomic DNA, terminase, or associated accessory viral proteins might be considered in innovative mechanistic studies.

## 3. Conclusions: Current State of the Art with Various NEC-Directed Investigational Options of Antiviral Interference

Combined, the ongoing investigation of the herpesviral nuclear egress and NEC composition and functionality clearly emphasize the rate-limiting importance of this stage of viral replication. Several groups have reported on their approaches to validate herpesviral NECs as highly suitable targets of novel antiviral strategies, some of which direct against core NEC formation or multicomponent association, including capsids, or against enzymatic activities linked to the NEC, such as the recently approved viral kinase inhibitor MBV. Due to the fact that herpesviral NEC functions combine a variety of complex PPI aspects and regulatory activities, as well as membrane- and transport-specific functions, the options of drug-mediated targeting are broad and diversified. In this review, several different targeting options and investigational tools of NEC inhibition were highlighted. These options were also discussed in terms of their applicability in the development of putative new NEC-directed antiviral agents. They include (i) the experimental validation of inhibitory effects provided by NEC protein mutants or modes of fragment overexpression, (ii) the specific drug discovery based on NEC-directed small molecules or synthetic peptides, (iii) the inhibition of enzymatic components of the core NEC-associated factors that provide multicomponent activities such as kinase inhibitors, and (iv) possibly additional modes of inhibition that have not yet been progressed to a defined translational stage. Specifically the latter option may include further NEC-related inhibitory targeting mechanisms, such as Pin1 inhibitors, blockers of capsid–NEC docking, or encapsidation-interfering agents, which appear highly interesting and challenging at the same time. One more, highly relevant, question is also the subject of current investigations, namely the option of generating a broadly acting, pan-antiherpesviral drug through targeting the nuclear egress. Such broad activity may be based, on the one hand, on the conservation of core NEC proteins and structures, and, on the other hand, on the interference with the diverse NEC-associated functional mechanisms. At present, it appears as an attractive, yet unproven, opportunity to achieve a pan-antiherpesviral potency through the action of NEC inhibitors. At least four points of current evidence argue for the realistic chances to realize this pan-antiherpesviral scenario based on NEC-inhibitory small molecules: (i) the 3D structures of herpesviral core NECs are proven to be strictly conserved; (ii) NEC functionality represents a rate-limiting step of herpesviral replication efficiency and pathogenesis; (iii) the association of NEC-specific regulatory and enzymatic factors, accessory to inhibitory drugs, appears to be a general property; and (iv) an experimental validation of the NEC as a suitable drug target has been achieved with several human and animal pathogenic herpesviruses. Principally, however, the NEC-directed antiviral activity might be realized in both ways, either in a virus-specific or a broad, pan-herpesviral manner. This aspect can be considered from two different points of view. As far as the structural conformity of the core NEC is concerned, one might see a chance to generate a broadly active NEC-directed inhibitor. On the other hand, the highly specific requirements towards a sterical hindrance of such small molecules have to be taken into greater account. Drug–target docking may be based on very specific interaction of the drug with individual amino acids, forming a binding pocket or a finely structured, exposed surface area (‘microcanyon’), which may argue against a broadness of inhibitory activity. This aspect may favor the idea that a virus-specific mode of NEC inhibition appears more probable than a broadly interfering mode, but further mechanistic investigations still have to answer this question. As a general note, NEC analyses and inhibitor developments are steadily ongoing on an intensive level, as driven by a number of international research groups. Thus, it can be expected that the proposed line of NEC-specific antiherpesviral targeting strategies will come to even more exciting results. Possibly, this may include an advanced translation-oriented and clinically applied outcome of drug discovery in the near future.
